# Challenges regarding the implementation of cervical cancer screening guidelines in Limpopo province, South Africa

**DOI:** 10.4102/phcfm.v16i1.4487

**Published:** 2024-07-30

**Authors:** Doris Ngambi, Dorah U. Ramathuba

**Affiliations:** 1 Department of Advanced Nursing Science, Faculty of Health Sciences, University of Venda, Sibasa, South Africa

**Keywords:** cervical cancer, challenges, professional nurses, implementation, public health institutions, policy, screening

## Abstract

**Background:**

The World Health Organization’s (WHO) call to eliminate cervical cancer is essential in improving structures and processes at primary healthcare facilities by galvanising change in providing cervical cancer screening services.

**Aim:**

The main objective of this study was to explore challenges affecting the implementation of cervical cancer screening guidelines in selected districts in Limpopo Province.

**Setting:**

The study was carried out at primary health care services (PHCs) in Vhembe and Mopani districts, Limpopo province.

**Methods:**

Qualitative non-experimental research design of an exploratory, descriptive and contextual nature of a qualitative paradigm to understand cervical cancer screening programme challenges from healthcare professionals’ perspectives. The study population comprised two males and 16 female professional registered nurses working in Limpopo province’s PHC services. The sample size was 18 professional nurses. A face-to-face interview guided by unstructured questionnaires was undertaken to elicit information regarding the implementation of cervical cancer screening services. Captured data were analysed using Tesch’s open-coding method.

**Results:**

The study revealed that the cervical cancer guidelines were not effectively implemented as there were contradictions and gaps when applying the guidelines about the management of HIV and AIDS, age restrictions and gestation. Furthermore, structural factors contributed to the inadequacy rate and failure to reach the set targets.

**Conclusion:**

Primary health care is an essential health care and human right; therefore, the government should ensure that guidelines and policies are supported financially and that professional nurses are capacitated for the efficient implementation of services.

**Contribution:**

Addressing the inequalities in the implementation of social policies for the prevention of cervical cancer prevention and improving the nurses’ knowledge and practice behaviour regarding cervical cancer prevention are imperative.

## Introduction

Cervical cancer is the fourth most common cancer among women worldwide, with the highest incidence in low-income countries (LICs), particularly in sub-Saharan Africa, with almost 90% of cervical deaths occurring in resource-constrained countries. Early cervical cancer screening can detect cervical intraepithelial grades 2 or 3 that are precancerous lesions and if detected, treated and managed early can prevent the development of invasive cervical cancer.^1,2^

The World Health Organization (WHO) has drafted a comprehensive global strategy built upon three main pillars: improving human papillomavirus (HPV) vaccination coverage, cervical cancer screening coverage and treating precancerous lesions. The WHO recommends a 5-year screening interval for women over 50 years and a 3-year interval for those within the age group of 25–49 years if resources are available.^3^

However, the guideline does not stipulate the frequency of screening for women below 25 years; the guideline is silent on the matter. The international guidelines vary; recently, guidelines from the United Kingdom recommend testing every 3 years for women aged 25–49 years but make no mention of what to do should the results not include endocervical cells. The American Cancer Society recommends annual repeat screening for women whose Pap smears show no endocervical cells, or earlier repeat testing for certain women. The guideline does not clarify which women qualify for early repeat testing; this ambiguity may cause confusion for physicians about who and when to retest.^4^ Tanzania, however, does not have a screening policy for cervical cancer; priorities are given to infectious diseases such as malaria, tuberculosis, diarrheal diseases, acute respiratory infections and sexually transmitted infections, all of which have individual control programmes.^5^

Cervical cancer screening was introduced in 2000 in South Africa. It was projected at a 70% coverage target by 2010; to date, 13.6% coverage has been achieved, and the mortality is still high.^6^ The SA national policy guideline on cervical cancer screening indicates that women with inadequate smears should be rescreened. If the repeat smear is also inadequate, the client must be referred to known competent screening services for better results.^7^ In the South African context, the cervical cancer policy and HPV vaccination programme were initiated in 2014, through the Integrated School Health Programme. The HPV vaccine is offered to girls aged nine to 12 years to primarily protect them against HPV types 16 and 18. All women 30–50 years should be screened once for cervical cancer within a period of 10 years, first at age 30 and then at 10-year intervals (i.e. at ages 40 and 50). Asymptomatic women under the age of 30 years should not be screened unless infected with HIV. Screening for HIV-positive women will be done irrespective of CD4 count and ARV treatment and continued at three-year or annual intervals. Women found with HG-SIL or CIN 2/3 will be offered appropriate pre-cancer treatment using ablative or excisional methods.^8^ Much still needs to be accomplished to enable South Africa to reach the 90-70-90 goal by 2030. The WHO Global strategy set three targets to be achieved by the year 2030 to get 90% of girls vaccinated with the HPV vaccine by age 15; 70% of women screened with a high-quality test by ages 35 and 45 and 90% of women with cervical disease receiving treatment.^9^

Screening women for precancerous lesions using the Papanicolaou (Pap) test has led to an average reduction of approximately 2.6% per year in cervical cancer mortality in countries with robust health systems.^10^ However, this approach has proven less effective in developing countries, mainly because of poor access to organised screening, low information on cervical cancer screening, women’s perception of a low threat of disease and overburdened, understaffed healthcare facilities that lack equipment and the need for laboratories.^11^ Nonetheless, significant gaps and challenges persist in reducing incidence and mortality and paving the way towards eliminating cervical cancer as a public health problem. The reality is that for most women in SA, screening services are either not available or either do not function effectively or are not accessed by those who need them, so this affects the utilisation of cervical cancer services negatively.^6^

## Research methods and design

### Study design

This study utilised a non-experimental research design of an exploratory, descriptive and contextual nature of a qualitative paradigm to understand cervical cancer screening programme challenges from healthcare professionals’ perspectives. Exploratory descriptive design was used to understand the challenges pertaining to the implementation of the cervical cancer screening programme. The study was also contextual as the phenomena of provision or implementation of cervical cancer screening programmes were studied directly from the perspectives of health care professionals within the context of their natural settings.

### Population and sampling

The study population comprised professional registered nurses working in primary health care (PHC) services in Limpopo province. A purposive sampling of professional nurses was sampled because they had the required characteristics for the study. A non-probability convenience sampling technique was used because it creates accessibility to participants that may be recruited for the study. The inclusion criteria were professional nurses who had worked in the clinics for over a year, rendered cervical cancer services and voluntarily agreed to participate. The sample size was 18 professional nurses and were all interviewed, though saturation occurred with participant 14. All participants were interviewed. The participants were accessed at Vhembe and Mopani district health PHC facilities in Limpopo province.

### Data collection method

Data collection is a procedure that is used by researchers when collecting information from participants to provide a contextual experience, revealing an experience as a process.^12^ An unstructured interview was the method used because it allows the researcher to understand the experiences of participants and the meaning they attach to their experiences. A face-to-face interview was undertaken to elicit information, expression of perceptions, attitudes and experiences from the participants concerning their working environment in the implementation of cervical cancer screening services. A pilot study was undertaken with two professional nurses to get a level of understanding of the interview question. The interviews were conducted in English, even though some participants mixed the conversation interview with their ethnic language (Venda or Tsonga). The researchers used a central question ‘What challenges do you experience in implementing cervical cancer screening services’? which were followed by probing questions that emerged from the data. Facilitation techniques were used to open communication and build rapport. The interviews were audio-recorded. Field notes describing the researchers’ observations and experiences during the interviews were recorded.

### Data analysis

Data analysis in qualitative studies begins during the interview process as the researcher is immersed in the data. During the interviews, the researcher wrote field short notes, which provided information needed in analysis, and the researcher could make connections and interrelationships among study data, which provided a rich context for analysis. Tesch’s method of data analysis was used.^12^ Transcriptions were read and reread to identify similar patterns. The identified themes and subthemes were coded and categorised according to similarities and relationships.

### Trustworthiness

Trustworthiness refers to the quality, authenticity and truthfulness of qualitative research findings. It relates to the degree of trust, or confidence readers have in the results.^12,13,14^ Credibility was achieved through the purposive sampling of participants, prolonged engagement, probing and paraphrasing of information from participants. A dense description of the research methods ensures transferability. Dependability was achieved through the independent coder, who also analysed the transcripts to achieve reliability, theme and subthemes and reached a consensus. Confirmability was achieved through audiotapes, transcripts and field note records.

### Ethical considerations

Ethics is a branch of philosophy that deals with decision-making concerning right and wrong.^12^ Adhering to ethical principles to protect research participants’ dignity, rights and welfare is essential. The researchers showed respect to participants by informing them about the purpose and processes of the study for them to make their own informed decisions about whether to participate or not participate in the research study. Participants were given information about the study and voluntarily consented (informed consent) before the study commenced; thereafter, written consent was signed. Confidentiality and anonymity are related to the rights of beneficence, respect for dignity and fidelity.^13^ Anonymity and confidentiality were ensured by ensuring that no personal identifiers were on the transcript and audiotape. Furthermore, the raw data were kept confidential under lock and key; no one had access to it, and the electronic data were password encrypted. Ethical clearance to conduct the study was obtained from the University Ethics Committee (SHS/19/PDC/08/1305) and the Provincial Department of Health of Limpopo province.

## Results

Table 1^15^ depicts a summary of the findings. The themes and subthemes are explained below, and participants’ quotes support all statements, and literature was done to verify the results.

**TABLE 1:** Themes and sub-themes.

Variable	Theme	Sub-theme
Challenges of health care professionals on the cervical cancer screening programme.	1. Poor uptake of the cervical programme.	1.1. Lack of interest or attitudes of health professionals. 1.2. Insufficient training for cervical cancer screening by district. 1.3. Culture as a barrier to screening. 1.4. Shortage of equipment for cervical cancer screening services.
Policy implementation and revision.	2. Concerns regarding the implementation of National cervical cancer Screening Policy.	2.1. Revision of the current policy. 2.2. Follow system.

*Source*: Ngambi D. Development of a training programme to strengthen cervical cancer screening in Limpopo province, South Africa [unpublished thesis]. University of Venda; 2021

### Biographic data

Eighteen healthcare professionals participated; 16 were females, and only two were males. The ages ranged from 35 to 60 years. Ten professional nurses had a degree in nursing and only eight had a diploma in nursing. The years of experience differed, with eight nurses having more than 15 years, three between 10 and 15 years and two between 5 and 10 years and five between 1 and 5 years. Generally, the participants were mature adults, and years of experience can be a determining factor in the practice and implementation of the cervical cancer screening programme.

#### Theme 1: Poor uptake of cervical cancer screening

Many women are diagnosed with cervical cancer at a stage when curative treatment is neither available nor possible. There is a need for commitment to and investment in training personnel, infrastructure and resources to complete cervical cancer screening pathways in reducing cervical cancer morbidity. Four subthemes emerged from the theme.

**Sub-theme 1.1: Lack of interest or attitudes of health professionals:** Cervical cancer screening was not implemented effectively because healthcare professionals are not displaying a positive attitude to women in the communities they serve, and the cervical cancer prevention and control programme is indeed important, resulting in poor uptake of the programme:

‘There are those client or patient who are not interested at all, yes who will move the client to the patient from point A to B to say please come tomorrow, today I do not have time I am all alone, please come tomorrow I do not have time, maybe it is because of the shortage of staff I do not know, but to me, it is not time-consuming.’ (Participant 5, female, 35 years, professional nurse)

‘I think it goes back to what I have said to say, that some nurses are not interested. Some clients themselves refuse to be screened by younger nurses. They prefer older nurses, and the older nurses are not interested, but otherwise, I think it is the reason we cannot reach 70% mmm …’ (Participant 8, female, 40 years, professional nurse)

Nurses play a significant role in enlightening the public on the availability and need for cervical cancer screening services. Their attitude is often crucial in gaining women’s confidence as they are the person who conduct the test.

**Sub-theme 1.2: Insufficient training for cervical cancer screening by health care professionals in districts:** Lack of standardised training and provision of on-site support is a challenge resulting in the low number of health care professionals providing cervical cancer screening services:

‘No, I was not trained, but my colleague showed me how to collect pap smear, but that was three years back, since then, no in-service training … I suggest that every professional nurse be trained before collecting pap smear.’ (Participant 7, female, 28 years, professional nurse)

‘I was working in the male medical ward; in the hospital, nobody is going to train you on how to collect it but only the doctors; they showed us how to do it, but also they were doing it anti-clockwise of the smear of which it is not allowed but the rest I have learned in the reproductive side yes.’ (Participant 10, female, 55 years, professional nurse)

‘It goes back to training; this thing was not part of the training; during the training, we were shown by our colleagues how to do the skill.’ (Participant 5, female, 35 years, professional nurse)

Batt et al.^16^ indicated that to promote up-to-date healthcare professionals’ competencies, health services managers play a critical role in ensuring that competency consolidation occurs in their services and is aligned with the principles of lifelong learning.

**Sub-theme 1.3: Culture as a barrier to screening:** Cancer diagnosis is a taboo, and it is associated with fear and death culturally. Most African black (race) women face cultural, emotional and practical barriers to doing pap smear tests because of the shame of examining the pelvic parts:

‘Like males, they do not agree to say you cannot let a woman lie and open her legs, it’s silly … So, if there is an older person, she will say no because this woman is older than me, how can I check in the private parts, it is so awkward.’ (Participant 9, female, 36 years, professional nurse)

‘[*M*]ale nurses do not have an interest when doing pap smears, and even the clinic manager, when your colleague has had such a challenge, we sometimes feel pity for him we will say they must not do this, but according to the guideline he had to do it, those are some of those barriers, as I have said the age of that patient sometimes the older women, you see … they will say ooh I can’t want to be seen by the younger daughter eeh … like that.’ (Participant 13, female, 52 years, professional nurse)

**Sub-theme 1.4: Lack or shortage of resources to implement cervical cancer screening:** The lack of resources seriously challenges healthcare professionals to render cervical cancer screening services. They sometimes find themselves in a dilemma when providing cervical cancer screening services.

‘Eeh … Not long ago, our machine was not working. Now it’s fixed. I think we can carry out the pap smears because if the machine is not working, we have to wait for the disposable cusco to be delivered, but if the cuscos are not delivered, we cannot carry out how will we do without cuscus.’ (Participant 6, female, 32 years, professional nurse)

‘Because the consumables are scarce, you can get it maybe after three months. The only thing is to take your car and visit a nearby clinic. Saying … Can you lend me five because you see that the month is ending? I need to do the statistics.’ (Participant 10, female, 55 years, professional nurse)

She added:

‘The pharmacists are trying their level best; the thing is up at the depot. After all, you can’t get all; maybe you can order a hundred and find that you have been given fifty, and the pharmacy will come and say borrow us for the other clinic does not have. Then you can’t say no.’ (Participant 10, female, 55 years, professional nurse)

She further added:

‘… shortage of resources and equipment; for example, we don’t have a sterilizer as we have to travel 20 km to sterilize our equipment.’ (Participant 10, female, 55 years, professional nurse)

The study conducted in Cape Town revealed that South Africa faces many challenges in implementing an effective cytology-based screening programme. Some challenges include human resource shortage, resource constraints, poorly functioning, health systems, competing health priorities and lack of access to health services, particularly for women with low socio-economic status living in rural or urban areas.^17^

**Sub-theme 1.5: Structural barriers (infrastructure):** Poor infrastructure and lack of privacy aggravated the unwillingness to provide cervical cancer screening.

Participants reflected on the following responses:

‘The infrastructure, for example, in the chronic where I am working is where we are seeing our HIV clients and find that I must do pap smears, and there is an examination coach, but there are no screening curtain for privacy, so the privacy is compromised and exposing the woman there you find that someone is knocking and without saying come in the person already came in.’ (Participant 4, female, 30 years, professional nurse)

‘Though we have the issue of privacy because we do not have a partition in the labour ward.’ (Participant 7, female, 26 years, professional nurse)

It has been noted that an accompanying lack of infrastructure, physical space and trained personnel to respond to these demands sometimes results in a barrier to quality cervical cancer screening services.

#### Theme 2: Concerns regarding the implementation of the National Cervical Cancer screening policy

Healthcare professionals are expected to adhere to guidelines to provide quality cervical cancer screening services at all levels of care.

**Sub-theme 2.1: Revision of the current policy:**

‘The cervical screening guideline is not clear, when to do, it is only the maternal guideline that says you must do, but the National cervical screening guideline does not include pregnant women. So, we end up not knowing whether we should do or not do these pregnant women.’ (Participant 5, female, 35 years, professional nurse)

‘It should indicate that we should screen every woman 30 years and above. If there is no problem, we should be repeating the pap smear after ten years, and we should screen those with a chronic condition, HIV and AIDS, so if we screen the patient like now, we should repeat the pap smear after three years and if there is a problem we should repeat the pap smear every year and the women should be screened three times for the whole life. No we have just browse it when doing in-service training.’ (Participant 7, female, 28 years, professional nurse)

‘Yes, we implement them, and we also give some of the clients who are educated, they ask to read, sometimes I photocopy those pages I think it is important then I give them, yes.’ (Participant 10, female, 55 years, professional nurse)

The findings generally indicated that understanding the policy was not a problem for health care professionals but challenging to practice or implement because of the contents of the policy that is questionable in some aspects, such as age restrictions and gestation, which requires some revision.

**Sub-theme 2.2: Follow-up system:** The potential reduction in morbidity and mortality through cancer screening cannot be realised without receiving appropriate follow-up care for abnormalities identified via screening.

‘It talks about … Those who are HIV positive, they have to repeat after one year, those who have typical squamous we do not have to keep them in the clinic have to transfer them to the hospital immediately.’ (Participant 10, female, 55 years, professional nurse)

Further added:

‘On that one, we will say [*laugh*] included or excluded but that patient has to be repeated in one year or six months some doctor that I was working with two years back and the reproductive consultant told us that those clients with HIV we are no longer going to wait for one year or six months because it can say a squamous when the cancer is already in situ.’ (Participant 10, female, 55 years, professional nurse)

Policy guideline provides recommendations for screen-and-treat strategies to prevent cervical cancer; however, these strategies should be reviewed constantly and made available at treatment centres for uniformity.

## Discussion

Cervical cancer is largely preventable through screening of adult women with prompt treatment of precancerous lesions. These interventions decrease cervical cancer morbidity when screening is performed well and with high population coverage. Findings from the study revealed poor uptake of the cervical cancer programme that is related to the lack of interest and the attitude of health professionals, as health professionals were not advising women who visited the clinic about cervical cancer screening services; limited health education was provided, while others felt that those who were provided with training should do the screening.

Negative attitudes of health professionals can be detrimental to the national strategic plan for screening and treating cervical cancer early in reducing the disease burden. Health services should provide the communities they serve with health information, provide community-based education and move from patient-centred care to community-based as there are community-based workers in rural communities. Fouka and Mantzorou^18^ indicate that medical science faces different challenges, and medical education should be moved from patient-centred towards being a community-based by designing educational programmes to improve health and understanding the health needs of society. This implies that all health professionals should be involved in providing cervical cancer screening. Obol et al.^19^ reported that 61% of participants who were not trained to conduct cervical cancer screening were more likely to have inadequate knowledge about cervical cancer than those who were trained. Umuago et al.^20^ and Tchounga et al.^21^ in their study conducted among health workers reported that attending training for cervical cancer prevention and treatment was significantly associated with an increase in knowledge. Furthermore, Obol et al.^19^ concurs with Umuago et al.^20^ that there is a need to increase the coverage of training for health workers about cervical cancer so that negative attitudes can be changed for them to be able to provide health education and promotion of cervical cancer screening and increase the uptake of the programme.

Participants in the study further iterated that there was insufficient training provided by the organisation, which was a barrier as a lack of knowledge and competency can hamper the uptake of cervical cancer screening programs. There are new advances in medicine and technology in the prevention of cervical cancer and nurses need to keep abreast with recent developments in prevention and treatment. The introduction of new prevention technology worldwide means that health professionals and managers need to understand the significance of new information, and they, in turn, must be able to transmit such information appropriately to communities with increased screening uptake. Company et al.^22^ indicate that health professionals often do not have many opportunities to update their knowledge and skills, and the same is true of other professionals such as nurses and public health professionals; however, new internet-based technologies have provided unique possibilities for medical training without the barriers of distance, time and space. Parra et al.^23^ indicated that low-cost universal cervical cancer instructional apparatus (LUCIA) is a low-cost excellent tool that can be utilised in rural areas to increase training capacity to screen, diagnose and treat precancerous cervical lesions based on local standard guidelines. Furthermore, Parra et al.^23^ indicate that after lecture sessions and hands-on teaching, telementoring sessions are encouraged to reinforce and amplify knowledge and skills.

The cultural factor was also indicated as a challenge to cervical cancer uptake because women were reluctant to be screened by male nurses or young nurses. Ndejjo et al.^24^ are in support of the need for more female staff to carry out screening because of the embarrassment felt by some when attended to by males and to have a mix of both younger and older staff to cater for all clients. Addressing culture-specific issues and provision of sensitive and competent services provided for the effective delivery of public health programmes.^25^

Challenges such as a lack or shortage of resources and infrastructure were also highlighted by participants as affecting the uptake of screening. Participants were concerned about patients’ safety and privacy. The majority of clinics did not receive an adequate number of speculums or disposable cuscus; the autoclaves were always out of order and respecting women’s privacy, and confidentiality was not upheld because nurses are using the same cubicles for consultations and all other medical procedures, and the communication could be overheard by other patients in waiting areas.

Black et al.^26^ also highlighted that lack of adequate health infrastructure and resources such as lack of speculum equipment were recognised as barriers to screening in Uganda, and women who presented for screening had to be turned away. Additionally, the importance of having a well-functioning health system is a constitutional right as health facilities should have adequate capacity and required supplies and logistics to provide quality screening services to women.^27^

The findings generally indicated that understanding the policy was not a problem to health care professionals but difficult to practice or implement because of the contents of the policy that is questionable on some aspects, such as age restrictions and gestation that required some revision. Sibiya^28^ indicated in a study conducted in KwaZulu-Natal that all high-grade squamous intra-epithelial lesions occurred in women younger than 30 years, which is much lower than the usual age distribution for high-grade lesions around 35–40 years, which is stated in cervical cancer screening policy. In South Africa, HIV and AIDS statistics are alarming; almost 8.5 million people are living with the virus, and almost a fourth of South African women in their reproductive age (15–49 years) are HIV positive, and an estimated 19.6% of the population are living with the virus.^29^ Screening guidelines have evolved rapidly, and many of the organisations that develop screening guidelines now agree to review the available evidence on cervical cancer screening and jointly produce new cervical cancer screening guidelines because of the greater understanding of the pathological development of cervical cancer and the discovery of the HPV DNA test and HPV vaccines that have occurred in the last decade.^30^ The main objective of the cervical cancer guideline is to decrease the incidence and prevalence of cervical cancer and any mortality associated with cervical cancer. To achieve this objective, individual countries must develop national policies, guidelines and protocols for cervical cancer prevention and control based on their own evidence.

The study findings highlighted a loss to follow-up because of poor coordination and lack of finances and infrastructure as women go missing in the system. The follow-up system is poor; women come to the public health facilities and are attended; however, their details are not complete, or at times they lose their phones and have new contact numbers and cannot be located telephonically, which poses a problem of secondary prevention and providing early treatment. At times, delays in the results and the false-negative test or poor cytology of pap smears contribute to loss of follow-up. The finding of the study is supported by Gago et al.^31^; who found that American Latina women with positive results failed to complete follow-up and treatment in most cases. The reasons cited were related to a deficient health services organisation, and subjective factors such as delay in result delivery or not receiving results at all were the most reported problem by women, followed by problems with appointment dates and long waiting times.

## Conclusion

The challenges of providing cervical cancer prevention were highlighted at the PHC facilities, which are the first point of contact care. It is at this level that proper education, screening for early detection, treatment and referral are made to reduce the burden of cervical cancer morbidity and mortality. Health workers’ knowledge and understanding about cervical cancer should be upscaled as it critically determines attitudes and behaviours towards cervical cancer prevention. Cervical cancer guidelines are to be implemented in relation to other guidelines.
